# Intraoperative methylene blue staining is effective as a single mapping technique in the identification of sentinel lymph nodes in dogs with low‐grade mast cell tumours

**DOI:** 10.1111/jsap.13832

**Published:** 2025-01-27

**Authors:** S. Zanardi, D. Guerra, S. Sabattini, A. Foglia, S. Del Magno, V. Cola, L. Pisoni, L. Ciammaichella, E. Faroni, C. Agnoli, A. Renzi, L. Marconato

**Affiliations:** ^1^ Department of Veterinary Medical Sciences University of Bologna Bologna Italy

## Abstract

**Objectives:**

The aim of this prospective study was to assess the association between methylene blue staining pattern and the presence of histologic nodal metastasis in dogs with low‐grade mast cell tumour in low‐resource settings for the efficient diagnosis of lymphatic spread.

**Methods:**

Dogs with a single, cytologically low‐grade mast cell tumour and no documented distant metastases were prospectively included and underwent surgery. Along with primary mast cell tumour removal, intraoperative sentinel lymph node mapping with peritumoral mast cell tumour injection and regional lymph node excision, regardless of whether blue dye was visible in the lymph node, were performed. Association between the lymph nodes with dye uptake (stained) and their metastatic status was evaluated.

**Results:**

Twenty‐five dogs were enrolled, and at least one stained lymph node was identified in 22 (88%) of them. A total of 49 lymphocentres were surgically inspected, and a total of 53 lymph nodes were removed. Twenty‐nine (54.7%) lymph nodes were stained, and 24 (45.3%) were unstained. Among the 29 stained lymph nodes, there were seven (24.1%) HN0, seven (24.1%) HN1, seven (24.1%) HN2 and eight (27.7%) HN3. Among the 24 unstained lymph nodes, 17 (70.8%) were HN0 and seven (29.2%) were HN1. No complications related to methylene blue injection were recorded.

**Clinical Significance:**

Peritumoral methylene blue injection is a cost‐effective alternative technique for detecting sentinel lymph node for dogs with mast cell tumours, particularly in economically constrained settings. All metastatic lymph nodes (HN2/HN3) were stained, and all unstained lymph nodes were non‐metastatic (HN0/HN1).

## INTRODUCTION

The lymphatic pathway is the primary route through which canine mast cell tumours (MCTs) metastasize, and the disease status of the sentinel lymph nodes (SLNs) is one of the most important prognostic factors, often guiding recommendations for adjuvant chemotherapy following surgical resection of the primary tumour along with lymphadenectomy (Blackwood et al., 2012; Ferrari et al., 2018; Marconato et al., 2020; Sabattini et al., 2021). Macroscopically, although severely enlarged lymph nodes (LNs) often are metastatic, LNs harbouring metastases are not necessarily enlarged (Ferrari et al., 2018).

Over the years, clinical practice has shifted towards initially removing the regional lymph node (RLN). More recently, attention has focused on SLNs rather than RLNs (Liptak & Boston 2019; Fournier et al., 2021). The SLN is defined as the first LN receiving the lymphatic drainage from the primary tumour, thereby also representing the first metastatic station. On the contrary, the RLN drains a specific anatomical area rather than the tumour itself (Suami et al., 2013). It has been documented that RLNs and SLNs do not match in up to 30% to 60% of cases (Annoni et al., 2023; Dogan et al., 2019; Ferrari et al., 2020 ; Ferraris et al., 2023). There are additional limitations to removing the RLN. First, the anatomic maps do not account for the individual variability, the overlap of several lymphocentres and the unpredictable nature of neoplastic lymphangiogenesis. Furthermore, since some lymphosomes or lymph basins consist of multiple LNs (Suami et al., 2013), the random extraction of a single LN may result in morbidity without providing any clinical benefit for the animal.

Unfortunately, SLN mapping is time‐consuming, costly and not always feasible, requiring preoperative and intraoperative techniques, the latter being useful in facilitating nodal detection during surgery. Preoperative mapping techniques include indirect lymphography (through x‐rays or computed tomography), contrast‐enhanced ultrasonography and lymphoscintigraphy (Annoni et al., 2023; Ferrari et al., 2020; Fournier et al., 2021).

Intraoperative techniques include the peritumoral injection of vital dyes [methylene blue (MB), or indocyanine green] or radioactive isotopes (technetium) (Ferrari et al., 2020). In humans, lymphoscintigraphy with technetium is considered the gold‐standard mapping technique for many cancers (Beer et al., 2018; Niebling et al., 2016). Each technique has its pros and cons in terms of availability, sensitivity, feasibility and costs, and SLNs identification varies among different preoperative mapping techniques (Annoni et al., 2023; Fournier et al., 2021; Hlusko et al., 2020).

Peritumoral injection of MB is a simple and cost‐effective technique. Allergic episodes and systemic effects, such as haemolytic anaemia, have been rarely reported in humans and cats, but not in dogs. Its usage is well‐documented in human medicine, also as a single technique, and its ease of application makes it a safe method, particularly in developing countries (Devarakonda et al., 2021; Halilbasic et al., 2021).

The aim of this prospective study was to assess the association between MB staining pattern and the presence of histologic nodal metastasis in dogs with low‐grade MCTs in low‐resource settings for the efficient diagnosis of lymphatic spread. This study was therefore done to offer a cost‐effective alternative to owners with financial constraints. We hypothesized that LNs with dye uptake (“stained”) would include both metastatic and non‐metastatic nodes, while those without dye uptake (“unstained”) would be histologically non‐metastatic.

## METHODS

The Ethical Committee of the University of Bologna approved the study (protocol number 137365 of May 23^rd^, 2023); written consent for entry into the study was obtained from all dog owners.

### Study design

This prospective clinical study was designed to assess the association between MB staining and presence of histologic nodal metastasis in dogs with low‐grade MCTs that underwent intraoperative lymphatic mapping following peritumoral injection of the dye.

Client‐owned dogs of any age, sex, weight and breed, with a single cutaneous, subcutaneous or mucosal low‐grade MCT were eligible for inclusion if owners declined preoperative lymphatic mapping but agreed to surgery.

To be enrolled, dogs were required to have no distant metastasis before surgery. The general health and tumour‐specific staging workup included a history and physical examination, complete blood cell counts with differential, serum biochemistry profile, coagulation profile, urinalysis, cytologic grading of the primary MCT indicative of a low‐grade tumour (Camus et al., 2016), thoracic radiographs, abdominal ultrasound and fine‐needle aspiration of liver and spleen, regardless of their sonographic appearance.

Based on the anatomic site of the primary MCT, RLNs were identified by consulting the lymphatic map (Suami et al., 2013) and then excised, regardless of the staining status of the LNs. If a lymphosome contained more than one LN, efforts were made to identify and remove all of them, both stained and unstained. In cases of ambiguous locations that could potentially drain to more than one RLN, LNs from different lymphocentres were removed.

Dogs were excluded from the study if they had multiple MCTs, underwent previous MCT excision and/or lymphadenectomy, had distant metastasis and/or cytologically high‐grade MCTs.

### SLN detection and surgical treatment

**FIG 1:**
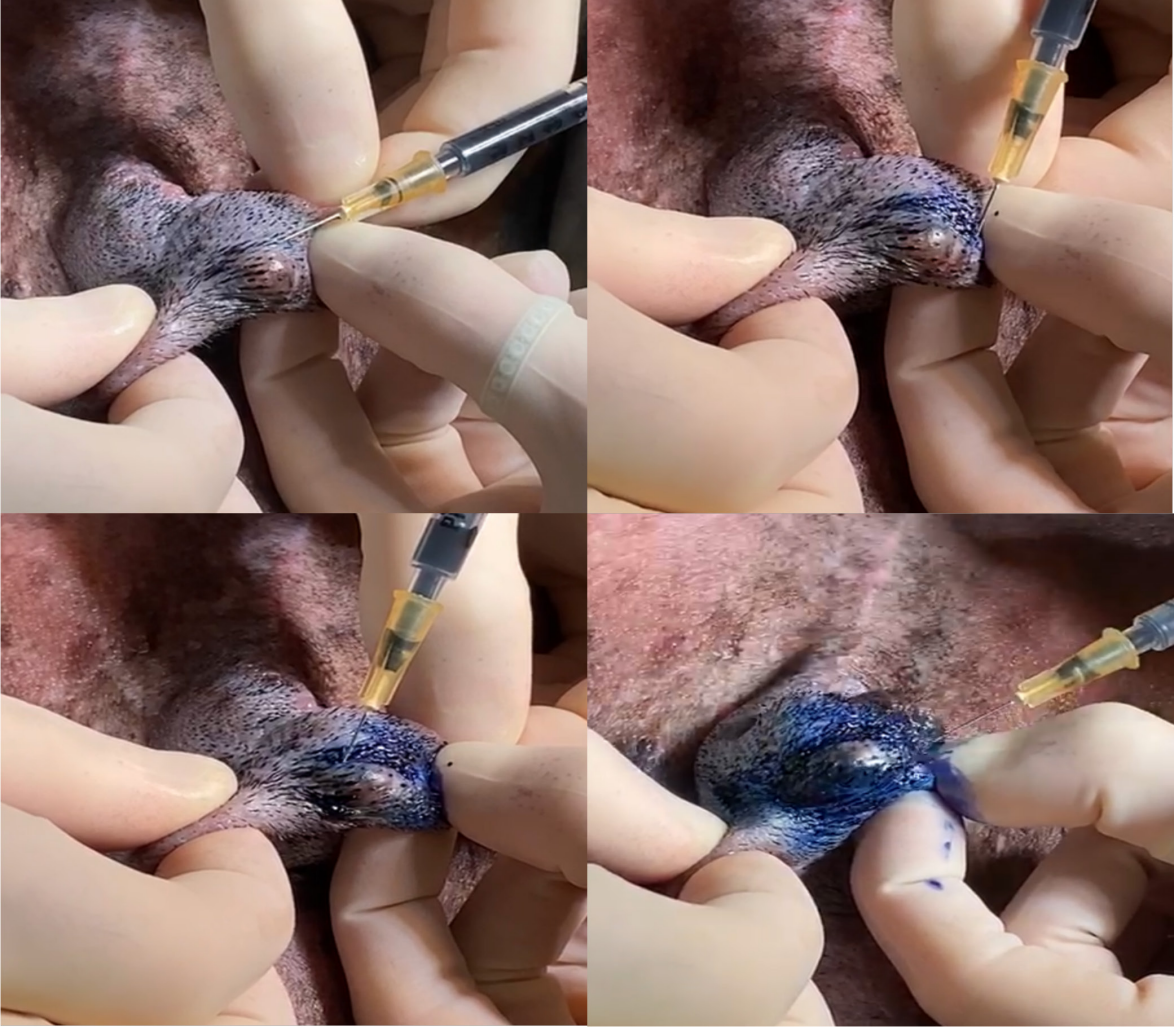
Case #1. Preoperative view of a MCT during peritumoral methylene blue injection at each cardinal point.

Within 10 to 14 days from the initial staging diagnostics, dogs were scheduled for intraoperative mapping and surgery, consisting of primary MCT removal and lymphadenectomy. While under general anaesthesia, a total volume of 0.4 mL (0.1 mL aliquots at each cardinal point), sterile 1% MB (SALF S.p.A; Cenate Sotto, Bergamo, Italy) was injected peritumorally using a 22 gauge needle syringe (Ferrari et al., 2020) (Fig 1).

Following MB injection, dogs underwent regional lymphadenectomy before MCT excision. The lymphocentres were surgically inspected and all identified LNs, stained and unstained, were removed.

**FIG 2:**
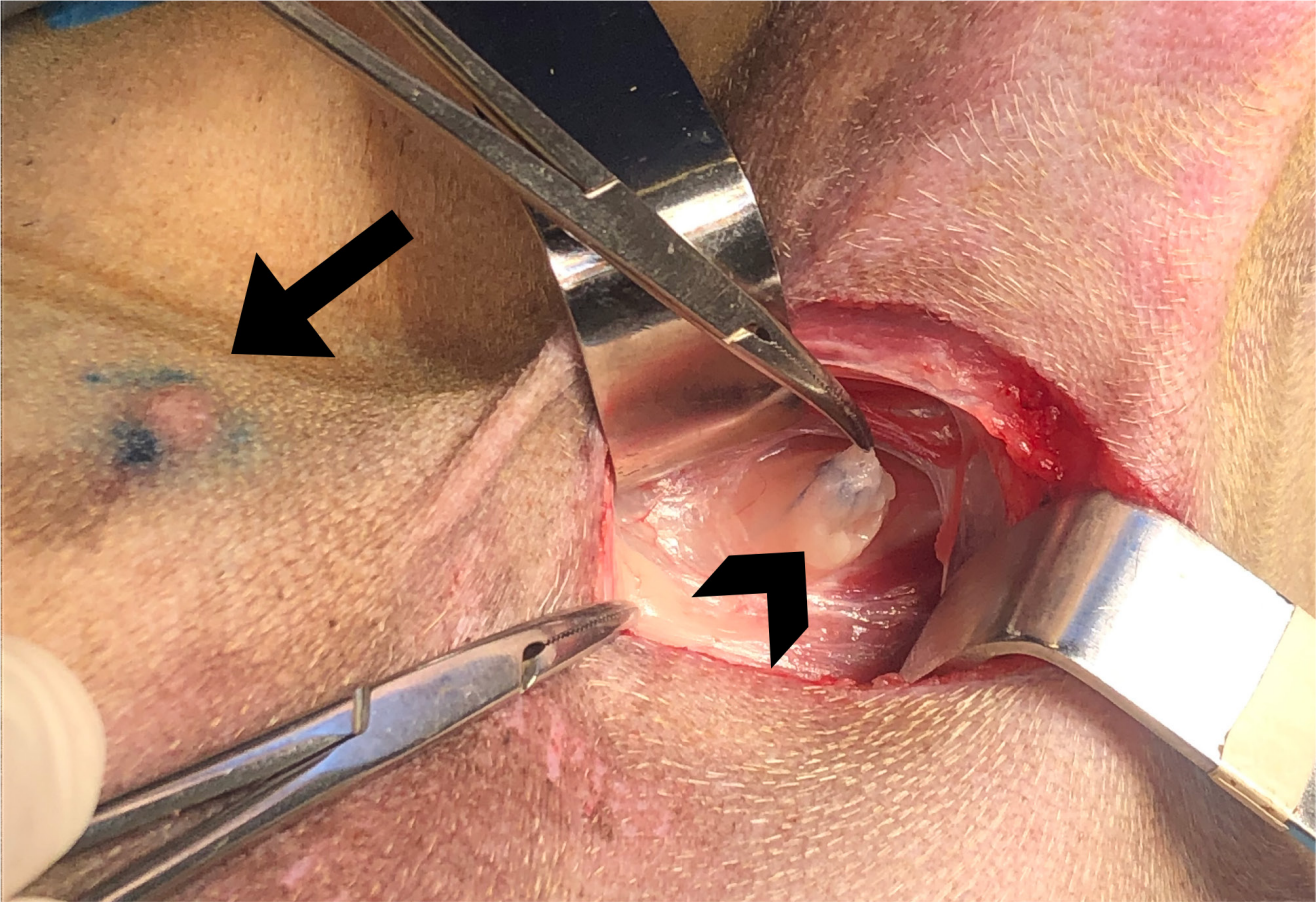
Case #18. Intraoperative view of the surgical approach to the axillary fossa. Note the blue colouring around the injected tumour area (arrow) and the stained LN (arrowhead).

The interval time between dye injection and LNs visualisation was recorded. LNs were defined as stained if blue dye was visibly present during gross visual inspection (Fig 2) and as unstained if no blue dye was visible.

Finally, MCTs were excised with curative intent, obtaining proportional margins for MCTs <2 cm and at least 2 cm of lateral margins for MCTs >2 cm, always ensuring a deep fascial plane.

Dogs were hospitalised for a minimum of 24 hours after surgery and followed up after 7 and 15 days. Any complications related to the injection of MB and/or to the surgery were recorded.

### Histopathology

The tumour and the excised LNs were histologically examined.

Samples were fixed in 10% neutral‐buffered formalin, processed and embedded in paraffin using a standardised protocol. Four‐micrometre‐thick histologic sections of the primary tumour, stained with haematoxylin and eosin, were microscopically examined for the assessment of histologic grade according to Kiupel and surgical margins (Kiupel et al., 2011).

Surgical margins were evaluated with combined radial and tangential sections and defined as complete (tumour cells >1 mm from the surgical margins) or incomplete (tumour cells ≤1 mm from the surgical margins) (Selmic & Ruple, 2020).

LNs were processed as previously described and stained with toluidine blue for the histologic node status evaluation according to Weishaar et al. (2014) and Sabattini et al. (2022).

All histologic evaluations were performed by a board‐certified veterinary pathologist (SS).

### Statistical analysis

Descriptive statistics were used in the analysis of dogs and tumour characteristics. When appropriate, data sets were tested for normality by use of the D'Agostino and Pearson omnibus test. No data had normal distribution, and all were therefore expressed as median (range).

Histopathologic reports were analysed for evidence of metastasis (HN2 and HN3) in stained and unstained LNs of each lymphocentre.

## RESULTS

### Dogs and tumour characteristics

A total of 25 dogs were enrolled in the study. The dogs and tumour characteristics are presented in Table 1.

**Table 1:** Summary of relevant patient and tumour characteristics observed in 25 dogs with mast cell tumours

Breeds	
Mixed‐breed	8 (32%)
Weimaraner	3 (12%)
French bulldog	3 (12%)
Labrador retriever	2 (8%)
Dachshund	2 (8%)
Golden retriever	1 (4%)
American Staffordshire bull terrier	1 (4%)
Boxer	1 (4%)
Shar Pei	1 (4%)
Oropa sheepdog	1 (4%)
Pug	1 (4%)
Great Dane	1 (4%)
Median age (range)	8 (4 to 13) years
Median weight (range)	20 (7 to 46) kg
Sex	
FS	12 (48%)
M	6 (24%)
NC	4 (16%)
FS	3 (12%)
MCT location	
Abdomen/flank	8 (32%)
Hindlimb	6 (4%)
Thorax	4 (16%)
Forelimb	2 (8%)
Perineal region	3 (12%)
Head and neck	2 (8%)
Median tumour diameter (range)	20 (1 to 100) mm
Ulceration	0 (0%)
Histopathologic growth pattern	
Cutaneous	13 (52%)
Subcutaneous	10 (40%)
Mucosal	2 (8%)
Histopathologic grade*	
Low‐grade	23 (100%)
High‐grade	0 (0%)
Surgical margins	
Complete	24 (96%)
Incomplete	1 (4%)

* Applied only to cutaneous and subcutaneous MCTs [*n* = 23; (Sabattini et al., 2024)]

M Male, F Female, NC Neutered male, FS Spayed female, MCT Mast cell tumour

### Lymph node mapping and lymphadenectomy

The median time interval between dye injection and nodal identification was 30 minutes (range, 10 to 120), with 30 minutes (range 10 to 70 minutes) for the stained and 39 minutes (range 10 to 120 minutes) for the unstained LNs.

No complications related to MB injection were recorded.

A total of 49 lymphocentres were surgically inspected, and a total of 53 LNs were removed, with a median of 2 LNs (range, 2 to 3) for each dog. Removed LNs included superficial inguinal (*n* = 23, 43.4%), popliteal (*n* = 8, 15.1%), deep axillary (*n* = 6, 11.3%), ventral superficial cervical (*n* = 5, 9.4%), mandibular (*n* = 5, 9.4%), hypogastric (*n* = 3, 5.7%), medial iliac (*n* = 2, 3.8%) and parotid (*n* = 1, 1.9%).

Twenty‐nine (54.7%) LNs were stained, and 24 (45.3%) were unstained. At least one (range, 1 to 2) stained LN was found in 22 (88%) dogs, whereas the staining of nodes was not appreciated in three (12%) dogs.

Among the 29 stained LNs, there were seven (24.1%) HN0, seven (24.1%) HN1, seven (24.1%) HN2 and eight (27.7%) HN3. Among the 24 unstained LNs, 17 (70.8%) were HN0 and seven (29.2%) were HN1.

The number and location of LNs, the time interval between dye injection and visualisation of the LNs and the corresponding nodal stage are listed in Table 2.

**Table 2:** Dogs, tumour and lymph node characteristics

Case	Signalment	Maximum diameter of the MCT (mm)	Location of the MCT	Number of excised LNs (number of excised lymphocentres)	Excised LNs	Stained/unstained	LN histologic status	Time of visualisation (min)
1	Dachshund M 12y 13 kg	10	Midline prepuce	2 (2)	Left SI	Stained	HN2	20
					Right SI	Unstained	HN0	20
2	Mixed breed FS 8y 7 kg	8	Left lower lip	3 (1)	Left SM	Stained	HN2	10
					Left SM	Unstained	HN1	10
					Left SM	Stained	HN2	10
3	Pug M 10y 9 kg	5	Left hindlimb	2 (2)	Left PO	Unstained	HN0	10
					Left SI	Stained	HN1	30
4	Dachshund M 10y 7.5 kg	3	Right inguinal	2 (2)	Right SI	Stained	HN0	31
					Right PO	Unstained	HN0	48
5	Oropa sheepdog MC 6y 25 kg	30	Midline prepuce	2 (2)	Right SI	Stained	HN3	60
					Left SI	Stained	HN3	65
6	Weimaraner FS 10y 22 kg	2	Right forelimb	2 (2)	Right AXE	Stained	HN0	95
					Right VCS	Stained	HN0	135
7	Mixed breed FS 6y 24 kg	10	Right cranial axillary fold	2 (2)	Right AXE	Stained	HN3	40
					Right VCS	Unstained	HN0	25
8	Mixed breed FS 10y 10 kg	30	Left hindlimb	2 (2)	Left PO	Unstained	HN0	60
					Left SI	Unstained	HN1	30
9	French bouledogue FS 10y 8 kg	10	Left hindlimb	2 (2)	Left PO	Stained	HN1	70
					Left SI	Stained	HN3	40
10	Weimaraner FS 5y 30 kg	20	Left flank	2 (1)	Left SI	Stained	HN1	55
					Left SI	Unstained	HN1	55
11	Shar‐pei FS 9y 25 kg	20	Right palmar carpal	2 (2)	Right AXE	Unstained	HN0	20
					Right VCS	Stained	HN3	40
12	French bouledogue MC 6y 25 kg	5	Left thorax (III nipple)	2 (2)	Left AXE	Stained	HN2	20
					Left SI	Unstained	HN0	30
13	Boxer M 12y 30 kg	5	Scrotum	2 (2)	Left SI	Stained	HN0	20
					Right SI	Unstained	HN0	30
14	American Staffordshire bull terrier FS 5y 15 kg	5	Left hindlimb	2 (2)	Left SI	Stained	HN1	25
					Left PO	Stained	HN0	40
15	Mixed breed MC 8y 10 kg	30	Right labial mucosa	3 (2)	Right SM	Stained	HN1	15
					Right SM	Unstained	HN1	30
					Right PA	Unstained	HN0	85
16	Mixed breed FS 7y 20 kg	100	Left stifle	2 (2)	Left SI	Stained	HN3	60
					Left PO	Unstained	HN0	120
17	Labrador retriever FS 8y 20 kg	25	Left caudo‐lateral antebrachium	2 (2)	Left AXE	Stained	HN3	20
					Left VCS	Unstained	HN0	110
18	Golden retriever F 7y 32 kg	16	Right thorax (IV rib)	2 (2)	Right AXE	Stained	HN3	20
					Right VCS	Unstained	HN1	50
19	Mixed breed FS 5y 18 kg	10	Right hindlimb	2 (2)	Right PO	Stained	HN1	60
					Right SI	Stained	HN2	35
20	Mixed breed FS 4y 7 kg	1	Midline prepuce	2 (2)	Right SI	Stained	HN0	25
					Left SI	Stained	HN0	20
21	Labrador retriever F 4y 34 kg	30	Tail	2 (2)	HY	Unstained	HN0	60
					Left MI	Unstained	HN1	70
22	Great Dane F 5y 45 kg	30	Right ischium	3 (3)	Right SI	Unstained	HN0	30
					Right MI	Unstained	HN0	90
					HY	Unstained	HN1	60
23	Mixed breed M 7y 16 kg	20	Right hindlimb	2 (2)	Right PO	Unstained	HN0	30
					Right SI	Stained	HN2	60
24	French bouledogue M 10y 13 kg	10	Scrotum	2 (2)	Right SI	Unstained	HN0	20
					Left SI	Stained	HN1	20
25	Weimaraner FS 10y 22 kg	20	Perineal	2 (2)	HY	Unstained	HN0	50
					Left SI	Stained	HN2	20

M Male, F Female, MC Neutered male, FS Spayed female, y Years, LNs Lymph nodes, SI Superficial inguinal, MI Medial iliac, SM Submandibular, PA Parotid, PO Popliteal, VCS Ventral cervical superficial, AXE Axillary, HY Hypogastric

Lymphedema was observed in seven (28%) dogs at the time of discharge, but it resolved spontaneously within 7 to 14 days postoperatively. No wound healing complications were documented.

## DISCUSSION

The present study demonstrates that MB dye can be effectively used alone for SLN mapping in low‐grade MCTs due to its cost‐effectiveness, excellent tolerability, and strong correlation with histologic findings in both stained and unstained LNs. This makes it a good option, especially in low‐resource settings. In this cohort of dogs, all unstained LNs were non‐metastatic (HN0/HN1) upon histologic examination, confirming our hypothesis. Among stained LNs, 15 were metastatic (HN2/HN3) and 14 were non‐metastatic (HN0/HN1).

A high percentage of dogs (88%) showed at least one stained LN, while in only three cases was the blue dye not observed in any of the explored lymphocentres. Notably, in two of these cases, the primary tumour was located in the upper lumbar region. After dye injection, the dogs were positioned supine to facilitate bilateral lymphadenectomy of the inguinal and medial iliac LNs. This position might have compressed the lymphatic vessels post‐dye injection, potentially reducing dye spread (Randall et al., 2020). The third dog received neoadjuvant chemotherapy before surgery to down‐size the tumour, which could have reduced dye uptake, as documented in human patients (Soebhi et al., 2020). However, due to the lack of a pre‐surgical detection technique, it is possible that the SLN was not exposed or removed.

Moreover, a similar rate (13%) of unidentified SLNs has been reported in human breast cancer, using MB as a single mapping technique (Halilbasic et al., 2021).

The use of the dye was advantageous for visualising LNs at the surgical site, potentially reducing the time needed for LN identification in this study. However, no significant differences were observed in the removal timing between the two groups. When a LN captured the dye, its colour contrast against the surrounding fat made it clearly visible, facilitating surgical dissection and delineation of LN boundaries. In some cases, the blue‐stained lymphatic vessels were clearly visible, allowing for tracking them to the LN. This observation may account for the differences in excision time between stained and unstained LNs.

During the surgical excision of the MCTs, the dye was visible in the peritumoral tissues in all dogs. It did not cause any intraoperative complications; however, the unusual coloration of the peritumoral tissues might obscure the differentiation between the muscular fascia and the subcutaneous tissue. Moreover, the dye injection could induce tissue swelling around the tumour, potentially blurring tumour demarcation, thereby complicating the establishment of surgical margins. However, despite these effects, the presence of the dye did not influence the histologic assessment of the margins. Complete excision was obtained in all dogs except one, and the percentage of incomplete margins aligns with previously reported data (Selmic & Ruple, 2020).

No episodes of allergic reactions or systemic responses were documented, confirming the safety of using this dye in dogs. Most of the MB injected was likely excised with the MCT, thereby reducing the risk of complications associated with the dye systemic absorption. Small amounts of MB might have remained in the surrounding areas of the surgical site or within the lymphatic vessels; however, no complications in wound healing have been documented, despite the possible incomplete removal of the dye from either the primary tumour or the LNs.

In seven dogs, lymphedema developed within 24 hours after surgery, possibly associated with multiple LNs dissections. Post‐operative lymphedema is already recognised as one of the most common complications following lymphadenectomy (Chiti et al., 2023).

There is no information in the literature regarding the time required for MB to be captured by the LNs. Therefore, it is possible that in unstained LNs, the dye might have diffused and exited the LNs before surgical dissection. However, in this study, all metastatic LNs were stained, and the median time for excision was 30 minutes. Therefore, it is plausible that the dye persists in metastatic LNs for at least 30 minutes.

A major limitation of this study is the absence of a preoperative mapping technique. As noted previously, it is possible that not all SLNs were removed; however, our results show that at least one SLN was identified in 88% of the dogs. In three cases, LNs were unstained. The lack of staining in these nodes should not be interpreted as evidence of a non‐metastatic status but rather as further confirmation that RLNs may not always serve as SLNs. This finding underscores the potential need for dissection of multiple lymphocentres, although such an approach would increase surgical time, costs, and patient morbidity.

The high rate of metastatic LNs corroborates the necessity of lymphadenectomy for accurate staging and treatment purposes, as the presence of residual neoplastic cells in the LNs can lead to disease progression (Marconato et al., 2018). Using this method, it is challenging to ascertain which LN first receives the initial lymphatic drainage, as more than one LN may share the lymphatic spread from a single mass, resulting in multiple stained LNs. The stained LNs are considered SLNs, and they may capture the blue dye even if they are non‐metastatic. Including dogs at different clinical stages could be a limitation in our study. However, it reflects the real‐world clinical scenario, where assessing LN status without histologic evaluation is impractical.

The identification of stained and unstained LNs was visually assessed without the use of objective tools, which could be considered another limitation of the study, as partially stained LNs might be visually overlooked (Ram et al., 2021).

In conclusion, the use of MB as single mapping technique allowed for the detection of at least one SLN in 88% of dogs with cutaneous, subcutaneous or mucosal low‐grade MCTs. Given the importance of lymphadenectomy and the high observed metastatic rate in stained LNs, excision of these nodes is recommended. Conversely, when at least one stained LN is identified, the excision of unstained LNs may potentially be avoided, as they have a low likelihood of metastases. This approach can help reduce potential post‐operative complications.

While SLN mapping with the combined use of preoperative and intraoperative techniques (e.g., radiographic or tomographic indirect lymphography) is always advisable, intraoperative MB staining alone offers a simple and cost‐effective alternative if the preoperative mapping cannot be pursued. This option may be particularly beneficial for owners facing financial constraints, even if it requires surgical access to multiple LNs.

It is worth noting that this technique may not be as effective for masses located in the upper lumbar region, where lymphatic vessels could be mechanically compressed or obstructed due to the patient's position during surgery, or in cases involving dogs undergoing neoadjuvant chemotherapy.

### Conflict of interest

None of the authors of this article has a financial or personal relationship with other people or organisations that could inappropriately influence or bias the content of the paper.

### Author contributions

**S. Zanardi:** Conceptualization (equal); data curation (equal); writing – original draft (equal). **D. Guerra:** Conceptualization (equal); data curation (equal). **S. Sabattini:** Analysis and interpretation of the data. **A. Foglia:** Data curation (equal); formal analysis (equal). **S. Del Magno:** Data curation (equal); writing – review and editing (equal). **V. Cola:** Data curation (equal). **L. Pisoni:** Data curation (equal). **L. Ciammaichella:** Data curation (equal). **E. Faroni:** Validation (equal). **C. Agnoli:** Validation (equal). **A. Renzi:** Formal analysis (equal); validation (equal). **L. Marconato:** Conceptualization (equal); project administration (equal); writing – review and editing (equal).

## Data Availability

The data that support the findings of this study are available from the corresponding author upon reasonable request.
